# The Effect of Transcranial Direct Current Stimulation (tDCS) on Resilience, Compassion Fatigue, Stress and Empathy in Professional Nurses

**DOI:** 10.9734/AIR/2015/16842

**Published:** 2015-06-08

**Authors:** Marietta P. Stanton, Rick A. Houser, Morgan E. Kiper Riechel, Joy J. Burnham, Graham McDougall

**Affiliations:** 1 Capstone College of Nursing, The University of Alabama, USA.; 2 College of Education, The University of Alabama, USA.

**Keywords:** Compassion fatigue, resilience, stress, transcranial direct current stimulation (tDCS), empathy

## Abstract

The purpose of the study is to determine the effect of Transcranial Direct Current Stimulation (tDCS) on measured levels of resilience and empathy in professional nurses with evidence of compassion fatigue and other stress related problems.

Lowered levels of resilience, compassion fatigue and decreased empathy are significant predictors of burnout in nurses. Enhanced levels of resilience are associated with improved empathic responses and overall emotional well-being. Nurses who work in high stress environments often exhibit compassion fatigue and post-traumatic stress disorders that may reduce their ability to function effectively. Because tDCS has been used successfully in a number of chronic disease conditions, it would seem that there is potential for it to be useful in a broader context. The treatment with tDCS may be a potential strategy for improving resilience and eliminating chronic stress responses.

A timed series counterbalanced research design was used for the study. Participants completed 18 sessions of tDCS over a six week period. They also completed a resilience, compassion fatigue, stress and empathy scale before and after each tDCS administration.

A repeated measure analysis was used to determine if tDCS had an impact on scale scores. The analysis showed that tDCS amperage had significant positive effects on empathy. On the outcomes of resilience, compassion fatigue and stress, tDCS did not produce any significant changes. This research provides a new approach to compassion fatigue, an old problem with caregivers. Notably, when implemented with individuals experiencing problems that involve apathy or indifference, tDCS is a non-effortful intervention that offers a pathway that may improve symptoms and does not require extensive outlays of physical or mental energy.

## 1. INTRODUCTION

The influence of compassion fatigue on nurses in various work settings has been noted with concern [1,2]. Coetzee and Klopper [3] found that compassion fatigue is a consequence of the stress felt by nurses and a function of their intense engagement with patients over time. Indicators associated with compassion fatigue include: apathy, fatigue, irritability, decreased productivity, boredom, diminished performance, an emotionally overwhelmed state, poor judgment, callousness, and desensitization to the needs of others [3,1]. In addition, Thompson [4] acknowledged that those who experience compassion fatigue struggle to provide good quality care to patients, while [5] concluded that stress and the ultimate burnout might diminish empathic responses from nurses.

With the symptoms related to compassion fatigue and the ensuing issues with diminished patient care, understanding and developing strategies to address compassion fatigue with nurses is crucial. Several studies have offered potential solutions for compassion fatigue. One approach was focused on promoting resilience in nurses [6,7]. Resilience has been described as the ability to adapt or bounce back following adversity and challenge and connotes inner strength, competence, optimism, flexibility, and the ability to cope effectively when faced with adversity [8,9]. Resilience was significantly associated with job satisfaction, reduction of stress, and even completion of a nursing program [8,9]. Additionally, mindfulness and other behavioral strategies may be used to increase resilience in nurses [6].

tDCS is an intervention that has been used in over 200 studies. tDCS and an expanding array of neurostimulation techniques in recent years has led to a greater understanding of functional anatomic relationships [10,11]. As an improved understanding of the underlying mechanisms of action for these emerging technologies has grown, the development of novel therapeutic interventions has been promoted. Due to its noninvasive nature and the utility of the technique, tDCS has been the focus of significant neuroscience research. In addition, the findings from studies that tested tDCS, have proposed mechanisms that may explain the development of neuroplasticity. tDCS has also been used as a treatment for neuropsychiatric conditions, specifically for the treatment of affective disorders such as depression and anxiety [12,13] and also within the neurological domain assisting in the motor rehabilitation of stroke patients [14]. The potential uses for tDCS with both remediation and enhancement are numerous [11]; however, the effects of tDCS on emotional states such as resilience, empathy, and compassion fatigue have not been examined.

Substantial research has been conducted on reintegration of nurses who experienced stress, compassion fatigue, and secondary traumatic stress syndrome in military nurse veterans [15]. However, many civilian nurses suffer from burnout and compassion fatigue. These conditions may impair their ability to effectively care for patients and make empathic decisions. Understanding the relationship between compassion fatigue, stress sequelae, resilience, and empathy is a critical need in this population. Using tDCS to enhance resilience may be a low cost, non-invasive, safe therapy for nurses who work in high stress environments or who care for patients in disaster or environments that promote hyperactive vigilance. Because tDCS has been used successfully in a number of chronic disease conditions, it would seem that there is potential for it to be useful in nurses with longstanding compassion fatigue and burnout.

The purpose of the study is to determine the effect of transcranial direct current stimulation (tDCS) on measured levels of resilience and empathy in professional nurses who have experienced compassion fatigue and symptoms of stress or burnout. Enhanced levels of resilience appear to improve empathic responses and the overall emotional well-being. tDCS may be a potential strategy for improving resilience and eliminating chronic stress responses.

Although there has been little or no research examining the impact of tDCS on resilience, compassion fatigue, and/or post stress symptoms in nurses, there are a number of studies that have examined the impact of tDCS on depression, anxiety, social cognition, anger modulation and emotional-affective aspects of pain [16,17].

There were two specific aims of this study. To determine: (1) If there were differences in before and after the administration of tDCS on compassion fatigue, stress, resilience, and empathic responses; and (2) If there was a differences in compassion fatigue, stress, resilience, and empathy based on the tDCS amperage that was delivered.

## 2. MATERIALS AND METHODS

### 2.1 Participants

Participants for this study were seven nurses who had worked in the hospital and/or other high volume care settings. They were between the ages of 30 −45 years old. There were six females and one male professional nurse. Some of the participants were staff nurses and several were nurse managers. Their educational level varied. Several of the nurses were masters prepared. The nurses were recruited from and worked in busy primary care clinics and emergency departments. The reason for using participants from such high volume care settings was that these nurses were most vulnerable to compassion fatigue and/or stress sequelae as well as compromised levels of resilience and empathy.

The following inclusion criteria were used for the participants in this study. Participants had to be working professional nurses who had worked full or part-time continuously for (a) a period of five years in a hospital or other high volume or acute care patient care institution; (b) may or may not have served in the military as a professional nurse within the past 10 years; (c) be medically cleared to participate in the study; and, (d) indicate that they felt overwhelmed or stressed in their current position.

Participants were excluded from participating in the survey if they (a) had any implanted medical device (i.e., pacemaker, defibrillator); (b) had a history of a metal brain implant or shunt; (c) had a history of severe head injury/neurosurgery; (d) had a history of fainting spells, seizures, or epilepsy; (e) had a history of stroke or heart attack; or (f) were pregnant.

### 2.2 Instruments

Four instruments were used in this study (see Table 1). The first instrument was the *Resilience Scale,* developed by [9], a well tested measure and has been used in a variety of situations. A meta-analysis of 12 different studies involving a variety of populations demonstrated that this scale can be used with a variety of populations equally well with excellent reliability and validity [18]. The second instrument was the [19] *Compassion Fatigue Scale*. This scale measured a secondary form of traumatic stress that is associated with caring for others. The third instrument, the *Perceived Stress Scale* [20] is the most widely used psychological instrument for measuring the perception of stress. This scale measures the degree to which situations in one’s life are appraised as stressful [21]. The fourth instrument was the *Empathy Assessment Index (EAI)* developed by Gerdes, Segal, and Lietz [22]. The EAI has five components: (1) affective response; (2) affective mentalizing; (3) self-other awareness; (4) perspective-taking; and (5) emotion regulation. The scale, which has 22 items, uses a Likert Scale ranging from 1 (never) to 6 (always). Two items (5 and 10) require reverse scoring.

Transcranial Direct Current Stimulation (tDCS) sends a constant low current when applied directly to the head partially penetrating the brain [23]. When the current passes through the anode (positive electrode) the neuronal long-term potentiation (LTP) increased the readiness of neurons for firing. Current passing through the cathode (negative electrode) decreases the neuronal, long term depression (LTD) and decreases the readiness of neurons for firing. Nitsche et al. [24] described the anodal (positive electrode) as increasing Long Term Potentiation (LTP) and resulting in neurons readiness for firing. Whereas, the cathode electrode has been described as decreasing readiness for firing (Long Term Depression, LTD. The low current was delivered via a Soterix Medical 1 X 1 tDCS Low-Intensity Simulator. The tDCS stimulator was set to administrator three levels of intensities of low current stimulation: 1.0 mA, 1.5 mA, and 2.0 mA (milliampheres). The Soterix Medical Stimulator is operated with two 9-volt batteries which delivers low level electrical current. The device provided a read out for true current (mA), the amount of current, and time of administration. There were four options for setting administration time for stimulation: 5 minutes, 10 minutes, 15 minutes, and 20 minutes. Twenty minutes of tDCS was used for the study. All direct current was administered to the scalp using a positive (anode) and a negative (cathode) electrode. Cells near the anode are stimulated by the positive electrical energy and activity in the cells in the area of cathode is diminished in response to negative electrical energy. The electrodes were encased in 5 cm × 7 cm (35 cm^2^) sponge pads. The sponge pads were moistened with 2–5 cc of saline solution. The electrodes and sponge pads based were placed on the scalp at two specific locations based upon the 10–20 international system for EEG electrode placement. Both electrode pads were held in place with head straps. The Soterix 1 × 1 stimulator had a control button for a sham condition when no electrical current was administered. The unit had a start and a shut-off button. Administration of the electrical current was ceased immediately by pushing the abort button.

A review of over 100 studies found no significant side effects associated with the administration of low electrical currents to the scalp [25]. Brunoni and colleagues [26] reviewed over 200 research studies using tDCS for adverse effects and found the following outcomes. Forty percent experienced itching (compared to 33 percent in a sham condition); 22 percent experienced tingling (18 percent in a sham condition); 15 percent experienced a headache (16 percent experienced a headache in the sham condition); and 8 percent experienced a mild burning sensation (10 percent of those in sham condition reported a burning sensation). All of these adverse reactions were temporary and did not result in participants withdrawing from the experimental condition.

### 2.3 Procedure

A flier was provided to registered nurses who lived and worked in the surrounding community that was targeted for this study. The recruitment flier gave a brief overview of the study and instructed potential subjects to contact the investigator by email or by phone. The investigators visited local nursing organizations and continuing education events and presented the study to groups of working nurses. It was made clear to potential participants that tDCS was an experimental treatment that had never been used with nurses and there was no documentation indicating that tDCS would be helpful. The participants were reminded that there was no direct benefit to them but that there was a possibility of a benefit, but this was unknown.

All potential subjects were interviewed to determine practice patterns as a professional nurse. Potential subjects had to provide documented medical clearance. Each participant completed a stress, resilience, empathy, and compassion fatigue scale prior to participating in the experiment. These are listed in Table 1. If the participant exhibited no stress or compassion fatigue on the scales prior to participation, they were not included in the experiment.

Based on recommendations from these previous studies, safety measures that have been used in other tDCS studies were incorporated into the protocol in this study [27]. During stimulation, all participants were requested to rate and indicate discomfort at the site of the electrodes or symptoms due to the stimulation. Ratings of scalp sensations were recorded every two minutes during tDCS administration using an 11-point scale where 0 indicated no sensation and 10 indicated a significant sensation (not tolerable). These were recorded during every tDCS session and stored with each individual’s scale measurements for that specific session.

To increase stimulation efficiency and limit subject anxiety, stimulus parameters such as current intensity, duration of stimulation, ramp up/down duration, stimulation mode, and impedance limit were programmed before the subject arrived. All electrodes and leads were plugged in and electrode sponges pre-moistened with a normal saline solution. Battery life was checked prior to each administration and changed to ensure no interruption of the procedure. The machine was placed out of view of the subject and all cables and wires were not allowed to obstruct the face during stimulation. A protocol and procedure manual was developed to guide the administration, evaluation, maintenance, and safety of the tDCS process. An appointed safety officer inspected procedures and records.

All procedures were reviewed and approved by the University of Alabama Institutional Review Board. All sessions were held in a secured lab or treatment area to protect the anonymity of participants. Each participant completed an informed consent prior to participation and all records were secured in a safe within a secure location accessible only by the research team.

### 2.4 Data Analyses

A total of seven nurses were administered tDCS three times per week for a total of six weeks. For the first and second week they were tested with 1.0 mA, for the third and fourth week they were tested with 1.5 mA, and for the fifth and sixth week they were tested with 2.0 mA stimulation. In each of the six weeks they were tested three different days, (i.e. Monday, Wednesday, and Friday) for each of the six weeks for a total of 18 times. Participants filled out questionnaires before and after each stimulation session. Each participant completed a total of 36 questionnaires. Due to the limitations of available participants no control group was possible.

The dependent variables included: resilience, compassion fatigue, perceived stress, and empathy. The objective was to investigate how responses to the four scales differed before and after amperage was administered as well as any change as the tDCS stimulation was increased to 1.0 mA, 1.5 mA, and finally 2.0 mA.

A timed series counterbalanced research design with three conditions was used for the study including: (1) Experimental Condition A which involved the use of stimulation at 1.0 mA current for 20 minutes; (2) Experimental Condition B which involved the use of 1.5 mA current for 20 minutes; and (3) Experimental Condition C which involved the use of 2.0 mA for 20 minutes.

Each condition, A; B; and C (counterbalanced), included three sessions each week (Monday, Wednesday, and Friday) for two weeks. After two weeks the condition was changed based on the counterbalanced design (Table 2). The participants completed the research in six weeks with two weeks allowed for each condition over three conditions. Previous researchers have found that tDCS is most effective with multiple administrations over a period of time [25]. This counterbalanced design was employed in a study by [16,17]. The rationale for using this design and the number of sessions in this current research was based on the definitive results these previous researchers achieved in their study using a similar approach.

For all three conditions, the cathodal electrode was placed over F2 and the anode was placed over T4 based on the 10–20 international system for EEG electrode placement. Measurements were completed on each participant and mapped on a predesigned tool to ensure proper placement of electrodes for each stimulation session. The placement of the cathode over F2 was to decrease stress response and compassion fatigue by decreasing neuronal firing. The anode was placed in the area of T4 which has been associated with empathic responses. Placing the cathode in this area was thought to stimulate empathic responses.

## 3. RESULTS

The initial analysis of the repeated measures for variables appears in Tables 3–7. SPSS uses a multivariate analysis to detect repeated measures effects. The lack of significance for the the p values shows that there was no significant interaction among the variables reflected by he repeated measures. All four multivariate tests did not reach statistical levels of significance. The analysis indicated a significant relationship between tDCS amp levels and the empathy scores. A post-hoc test (Benferroni) was used to compare every possible combination. This test controlled Type I error rate and kept the experiment-wise error rate to a fixed limit. No significance was found for the repeated measurements and administration of the tDCS over time, during any of the 18 sessions, and for any of the variables: resilience, compassion fatigue, perceived stress, and empathy included in the studies.

A one way analysis of variance (ANOVA) was also used employing the softwares JMP and SPSS. The usual assumption of independence, normality and constant variance were more or less satisfied. The response variables were resilience, compassion fatigue, perceived stress and empathy. The tDCS amp was the categorical predictor variable which had three levels 1.0, 1.5 and 2.0 amps. For each response variable, the average score of all the seven participants as the observation point was used. There were 12 observation points in each level totaling 36 observations. The results are summarized in the next section.

The amperage of tDCS accounted for about 24.59% of the variability in the empathy scores (p-value 0.0095, Table 6). The Benferroni test discussed earlier indicated that there were significant differences in the mean levels between 1.0 and 1.5 amp (p-value .055) and 1.0 and 2.0 amp (p-value .012). No significant difference was rated between the 1.5 and 2.0 amp levels.

The impact of tDCS amps on resilience was the first variable analyzed. Change in the tDCS amp (i.e. 1.0, 1.5 and 2.0 amps) explained only 3.12% in the variability of the resilience score, which indicated a weak relationship between tDCS amp and resilience scores (p-value 0.59, Table 7. tDCS amps explained only 4.57% in the variability of compassion fatigue score, which indicated a weak relationship between tDCS amps and compassion fatigue (p-value 0.46, Table 8). tDCS amps explained only 7.23% in the variability of perceived stress score, which indicated a weak relationship between tDCS amps and perceived stress (p-value 0.289, Table 9).

There were two specific aims of this study. To determine: (1) If there were differences in before and after the administration of tDCS on compassion fatigue, stress, resilience, and empathic responses; and (2) If there was a differences in compassion fatigue, stress, resilience, and empathy based on the tDCS amperage that was delivered. Results indicate that there were no differences before and after the administration of tDCS on scores on resilience, compassion fatigue, stress and empathy scales and there were no relationships between and among any of the variables. So the answer to research question one is no. As for question two, there appears to be a significant relationship between the tDCS amps used and the scores achieved on the empathy scale (See Table 10). So the answer to question two is no to all variables except empathy.

## 4. DISCUSSION

This pilot study contributes new knowledge to the literature on the treatment of stress and burnout in professional nurses. This novel treatment may offer relief to caregivers who are facing compassion fatigue and burnout from chronic stress. Nurses are leaving the profession in large numbers and many reported burnout and compassion fatigue as instrumental reasons for their decision [1,3,28,4]. Health care organizations are struggling with major shortages of nurses, staffing problems, and adjusting to consolidation of resources.

Based on the evidence in the literature, it appears that tDCS has the potential to decrease stress responses and perhaps mitigate compassion fatigue over time [7,8]. tDCS has been used in the treatment of affective disorders such as depression and anxiety [12,13]. tDCS also had a positive relationship with resilience and can stimulate empathic response [6]. It also appears that the tDCS Amperage has a significant relationship with Empathy. Although some participants informally reported noticeable changes in their resilience level or compassion fatigue, stress, and empathic responses, there were no significant changes according to the scale scores. The only change was noted in empathy albeit non-significant until the amperage was increased to 2.0. This finding may have ramifications for future study.

Common across all seven participants was the frustration and fatigue with completing the questionnaires before and after each session. In future testing, it would not be recommended to collect data before and after each session, but perhaps at the beginning and end of sessions when the amperage changes rather than every time. None of the participants complained of any local or systemic reactions to the procedure. Most of the subjects reported they “liked’ or “feel better’ after a tDCS session. Some even state that they “enjoyed” the sessions. However, they were adamant that the three days a week schedule of the study was wearisome and the repetitive paperwork diminished their responses.

Anecdotally, as one reviews the general informal notations made by the principal investigator, there was a more intense response to tDCS in those nurses who came to the sessions appearing agitated. They appeared to receive more relief from their agitation. The majority of respondents were female. One participant had a heightened positive response and indeed asked if they could buy the machine at the conclusion of the research they liked the sessions so much.

There were limitations to this study. A major limitation was the small sample size. Also, generalizability must be questioned. Basing conclusions about the impact of tDCS on empathy (and other dependent measures) using traditional paper and pencil measures may not be valid. The networks of the brain may not be located in one single area of the brain, but utilize combined networks of different areas and may require more complex and sensitive assessment and measurement to determine exact location of the response. Therefore, examining relationships between and among psychological variables and tDCS using conventional paper and pencil measures may be difficult.

The self-report nature of the dependent measures may also raise issues. Participants who function at higher levels of cognitive development are more likely to be self-aware and self-report lower levels of empathy than those at lower levels of cognitive development Gerdes et al. [5]. Cognitive development appears related to levels of responsibility, decision-making and leadership as a professional—those who were more agitated and intense at work were perhaps experiencing higher levels of responsibility.

There were recommendations based on this study. The study should be completed with a larger sample and be streamlined so that there is not repetitive survey completion during the process. It is also recommended that the amps be set at 2 since the best responses occurred at that amperage. Other physiologic measures like vital signs, cortisol levels (before and after) and EEG monitoring during tDCS sessions may also help to identify changes occurring as a result of tDCS and be more sensitive to changes that may not be captured by a paper and pencil tests. It is also recommended that tDCS be compared to mindfulness and other complementary and alternative stress relief activities and that it be studied alone as well as in conjunction with other stress relief strategies.

## 5. CONCLUSION

Results indicate that there were no differences before and after the administration of tDCS on scores on resilience, compassion fatigue, stress and empathy scales and there were no relationships between and among any of the variables.here appears to be a significant relationship between the tDCS amps used and the scores achieved on the empathy scale.

## Figures and Tables

**Table 1.** Instruments used in study

Scale	Concept measured	Number of items	Published reliability scores	Source
Resilience	Resilience	25	>.54-.75	Wagnild 2009 [9]
Compassion fatigue	Compassion fatigue	15	>.87	Figley 1995 [19]
Perceived stress	Post deployment trauma	10	>.78	Cohen and Williamson 1988 [21]
Empathy assessment index	Measures of empathy	22	>.91	Gerdes, Segal, and Lietz 2012 [5]

**Table 2.** Single-subject counter-balanced design

Participant	Condition	Condition	Condition
1	A (1.0 mA)	B (1.5 mA)	C (2.0 mA)
2	A (1.0 mA)	C (2.0 mA)	B (1.5 mA)
3	B (1.5 mA)	A (1.0 mA)	C (2.0 mA)
4	B (1.5 mA)	C (2.0 mA)	A (1.0 mA)
5	C (2.0 mA)	A (1.0 mA)	B (1.5 mA)
6	C (2.0 mA)	B (1.5 mA)	A (1.0 mA)
7	C (2.0 mA)	B (1.5 mA)	A (1.0 mA)

**Table 3.** Repeated measures over 18 sessions (resilience)

Tests of within-subjects effects						
Measure	Source	Type III sum of squares	df	Mean squares	F	Sig
Factor1	Sphericity Assumed	673.317	17	39.607	.865	.616
	Greenhouse-Geisser	673.317	1.985	339.138	865	.433
	Huynh-Feldt	673.317	2.576	261.405	865	.456
	Lower bound	673.317	1.000	673.317	865	.371
Factor1*group	Sphericity Assumed	162.571	17	9.563	.209	1.0
	Greenhouse-Geisser	162.571	1.985	81.884	.209	.812
	Huynh-Feldt	162.571	2.576	63.116	.209	.863
	Lower bound	162.571	1.000	162.571	.209	.656
Error(factor1)	Sphericity Assumed	9344.111	204	45.804		
	Greenhouse-Geisser	9344.111	23.825	392.205		
	Huynh-Feldt	9344.111	30.909	302.309		
	Lower bound	9344.111	12.00	778.676		

Computed using alpha = .05

**Table 4.** Repeated measures over 18 sessions (compassion fatigue)

Tests of within-subjects effects						
Measure	Source	Type III sum of squares	df	Mean squares	F	Sig
Factor1	Sphericity Assumed	157.750	17	9.279	.926	.544
	Greenhouse-Geisser	157.750	4.732	33.335	.926	.467
	Huynh-Feldt	157.750	8.841	17.843	.926	.505
	Lower bound	157.750	1.000	157.750	.926	.355
Factor1*group	Sphericity Assumed	48.258	17	2.839	.283	.998
	Greenhouse-Geisser	48.258	4.732	10.197	.283	.913
	Huynh-Feldt	48.258	8.841	5.458	.283	.977
	Lower bound	48.258	1.000	48.258	.283	.604
Error(factor1)	Sphericity Assumed	2044.270	204	10.021		
	Greenhouse-Geisser	2044.270	56.788	35.998		
	Huynh-Feldt	2044.270	106.091	19.269		
	Lower bound	2044.270	12.00	170.356		

Computed using alpha = .05

**Table 5.** Repeated measures over 18 sessions (perceived stress)

Tests of within-subjects effects						
Measure	Source	Type III sum of squares	df	Mean squares	F	Sig
Factor1	Sphericity Assumed	55.984	17	3.293	.821	.667
	Greenhouse-Geisser	55.984	3.816	14.673	.821	
	Huynh-Feldt	55.984	6.282	8.911	.821	.562
	Lower bound	55.984	1.000	55.984	.821	.383
Factor1*group	Sphericity Assumed	23.556	17	1.386	.346	
	Greenhouse-Geisser	23.556	3.816	6.174	.346	.837
	Huynh-Feldt	23.556	6.282	3.749	.346	.917
	Lower bound	23.556	1.000	23.556	.346	.568
Error(factor1)	Sphericity Assumed	818.016	204	4.010		
	Greenhouse-Geisser	818.016	45.787	17.866		
	Huynh-Feldt	818.016	75.389	10.851		
	Lower bound	818.016	12.00	68.168		

Computed using alpha = .05

**Table 6.** Repeated measures over 18 sessions (empathy)

Tests of within-subjects effects						
Measure	Source	Type III sum of squares	df	Mean squares	F	Sig
Factor1	Sphericity Assumed	1025.857	17	60.345	.696	.805
	Greenhouse-Geisser	1025.857	1.911	536.728	.696	.503
	Huynh-Feldt	1025.857	2.454	418.021	.696	.535
	Lower bound	1025.857	1.000	1025.857	.696	.420
Factor1*group	Sphericity Assumed	191.175	17	11.246	.130	1.0
	Greenhouse-Geisser	191.175	1.911	100.022	.130	.870
	Huynh-Feldt	191.175	2.454	77.901	.130	.914
	Lower bound	191.175	1.000	191.175	.130	.725
Error(factor1)	Sphericity Assumed	17687.968	204	86.706		
	Greenhouse-Geisser	17687.968	22.936	771.195		
	Huynh-Feldt	17687.968	29.449	600.631		
	Lower bound	17687.968	12.00	1473.997		

Computed using alpha = .05

**Table 7.** ANOVA (response variable: resilience and predictor variable: tDCS amp)

Source	DF	Sum of squares	Mean square	F ratio	Prob > F
Amp.	2	4.29824	2.14912	0.5314	0.5927
Error	33	133.46659	4.04444		
Total	35	137.76483			

**Table 8.** ANOVA (response variable: compassion fatigue, and predictor variable: tDCS amp)

Source	DF	Sum of squares	Mean square	F ratio	Prob > F
Amp.	2	1.347406	0.673703	0.7903	0.4621
Error	33	28.130450	0.852438		
Total	35	29.477856			

**Table 9.** ANOVA (response variable: perceived stress and predictor variable: tDCS amp)

Source	DF	Sum of squares	Mean square	F ratio	Prob > F
Amp.	2	0.821850	0.410925	1.2863	0.2898
Error	33	10.542025	0.319455		
Total	35	11.363875			

**Table 10.** ANOVA (response variable: empathy, and predictor variable: tDCS amp)

Source	DF	Sum of squares	Mean square	F ratio	Prob > F
Amp.	2	46.06382	23.0319	5.3811	0.0095*
Error	33	141.24468	4.2801		
Total	35	187.30850			

## References

[1] Maiden J, Georges JM, Connelly CD. Moral distress, compassion fatigue, and perceptions about medication errors in certified critical care nurses. Dimensions of Critical Care Nursing. 2011;30(6):339–345. 21983510 10.1097/DCC.0b013e31822fab2a

[2] Neville K, Cole DA. The relationships among health promotion behaviors, compassion fatigue, burnout, and compassion satisfaction in nurses practicing in a community medical center. Journal of Nursing Administration. 2013; 43(6):348–354. 23708503 10.1097/NNA.0b013e3182942c23

[3] Coetzee SK, Klopper HC. Compassion fatigue within nursing practice: A concept analysis. Nursing & Health Sciences. 2010; 12(2):235–243. 20602697 10.1111/j.1442-2018.2010.00526.x

[4] Thompson A How Schwartz rounds can be used to combat compassion fatigue: Alison Thompson reflects on an initiative that helps to maintain staff wellbeing and quality of patient care. Nursing Management. 2013;20(4):16–20. 10.7748/nm2013.07.20.4.16.e1102 23923178

[5] Gerdes KE, Geiger JM, Lietz CA, Wagaman MA, Segal EA. Examination of known-groups validity for the Empathy Assessment Index (EAI): Differences in EAI scores between social service providers and service recipients. Journal of the Society for Social Work and Research. 2012;3(2):94–112.

[6] Foureur M, Besley K, Burton G, Yu N, Crisp J. Enhancing the resilience of nurses and midwives: Pilot of a mindfulness-based program for increased health, sense of coherence and decreased depression, anxiety and stress. Contemporary Nurse. 2013;45(1):114–125. 24099232 10.5172/conu.2013.45.1.114

[7] Hodges HF, Keeley AC, Troyan PJ. Professional resilience in baccalaureate-prepared acute care nurses: First steps. Nursing Education Perspectives. 2008; 29(2):80–89. 18459622 10.1097/00024776-200803000-00008

[8] Matos PS, Neushotz LA, Griffin MTQ, Fitzpatrick JJ. An exploratory study of resilience and job satisfaction among psychiatric nurses working in inpatient units. International Journal of Mental Health Nursing. 2010;19(5):307–312. 20887604 10.1111/j.1447-0349.2010.00690.x

[9] Wagnild GM, Collins JA. Assessing resilience. Journal of Psychosocial Nursing and Mental Health Services. 2009;47(12): 28–33. 10.3928/02793695-20091103-01 20000280

[10] Dubljevic V, Saigle V, Racine E. The rising tide of tDCS in the media and academic literature. Neuron. 2014;82(4):731–736. 24853934 10.1016/j.neuron.2014.05.003

[11] Kadosh R The stimulated brain: Cognitive enhancement using non-invasive brain stimulation.(ed). Waltham, MA: Academic Press; 2014.

[12] Mondino M, Bennabi D, Poulet E, Galvao F, Brunelin J, Haffen E. Can transcranial direct current stimulation (tDCS) alleviate symptoms and improve cognition in psychiatric disorders? World Journal Biological Psychiatry. 2014;15(4):261–274. 10.3109/15622975.2013.876514 24447054

[13] Murphy DN, Boggio P, Fregni F. Transcranial direct current stimulation as a therapeutic tool for the treatment of major depression: Insights from past and recent clinical studies. Current opinion in psychiatry. 2009;22(3);306–311. 19339889 10.1097/YCO.0b013e32832a133f

[14] Hummel F, Cohen LG. Improvement of motor function with noninvasive cortical stimulation in a patient with chronic stroke. Neurorehabilitation and Neural Repair. 2005;19(1):14–19. 15673839 10.1177/1545968304272698

[15] Stanton MP, Dittmar SS, Jezewski MA, Dickerson SS. Shared experiences and meanings of military nurse veterans. Image: The Journal of Nursing Scholarship. 1996;28(4):343–347. 10.1111/j.1547-5069.1996.tb00385.x 8987282

[16] Boggio PS, Zaghi S, Fregni F. Modulation of emotions associated with images of human pain using anodal transcranial direct current stimulation (tDCS). Neuropsychologia. 2009;47(1):212–217. 18725237 10.1016/j.neuropsychologia.2008.07.022

[17] Palm U, Schiller C, Fintescu Z, Obermeier M, Keeser D, Reisinger E, Padberg F. Transcranial direct current stimulation in treatment resistant depression: A randomized double-blind, placebo-controlled study. Brain Stimulation. 2012; 5(3):242–251. 21962978 10.1016/j.brs.2011.08.005

[18] Wagnild G A review of the resilience scale. Journal of Nursing Measurement. 2009;17(2):105–113. 19711709 10.1891/1061-3749.17.2.105

[19] Figley CR. (Ed.). Compassion fatigue: Coping with secondary traumatic stress disorder in those who treat the traumatized (No. 23). Psychology Press; 1995.

[20] Cohen S, Kessler RC, Gordon LU. Strategies for measuring stress in studies of psychiatric and physical disorders. Measuring stress: A Guide for Health and Social Scientists. 1995;3–26.

[21] Cohen S, Williamson G. Perceived stress in a probability sample of the US Spacapam S, Oskamp YS (Eds.): The social psychology of health: Claremont Symposium on Applied Social Psychology. 1988;31–67.

[22] Gerdes KE, Segal EA, Lietz CA. Conceptualizing and measuring empathy. British Journal of Social Work. 2010;40(7): 2326–2343.

[23] Moreno-Duarte l, Gebodh N, Schestatsky P, Guleyupoglu B, Rato D, Bison M, Fregni. Transcranial electrical stimulation: Transcranial direct current stimulation (tDCS), Transcranial alternating current stimulation (tACS), transcranial pulsed current stimulation (tDCS, and transcranial random noice stimulation (tRNS) In Kadosh R (Ed.). The stimulated brain: Cognitive enhancement using non-invasive brain stimulation. Waltham, MA: Academic Press; 2014.

[24] Nitsche M, Doemkes S, Karakose T, Antal A, Liebetanz D, Lang N, Tergau F, Paulus W. Shaping the effects of transcranial direct current stimulation on the human motor cortex. Journal of Neurophysiology. 2007;97:3109–3117. 17251360 10.1152/jn.01312.2006

[25] Stagg CJ, Nitsche MA. Physiological basis of transcranial direct current stimulation. The Neuroscientist. 2011;17(1):37–53. 21343407 10.1177/1073858410386614

[26] Brunoni AR, Amadera J, Berbel B, Volz MS, Rizzerio BG, Fregni F. A systematic review on reporting and assessment of adverse effects associated with transcranial direct current stimulation. The International Journal of Neuropsycho-pharmacology. 2011;14(08): 1133–1145. 10.1017/S1461145710001690 21320389

[27] Borckardt JJ, Bikson M, Frohman H, Reeves ST, Datta A, Bansal V, George MS. A pilot study of the tolerability and effects of high-definition transcranial direct current stimulation (HD-tDCS) on pain perception. The Journal of Pain. 2012; 13(2):112–120. 22104190 10.1016/j.jpain.2011.07.001

[28] Neville K, Cole DA. The relationships among health promotion behaviors, compassion fatigue, burnout, and compassion satisfaction in nurses practicing in a community medical center. Journal of Nursing Administration. 2013; 43(6):348–354. 23708503 10.1097/NNA.0b013e3182942c23

